# Embolic Stroke and Meningitis Secondary to *Staphylococcus lugdunensis* Native Valve Endocarditis

**DOI:** 10.1155/2019/7910262

**Published:** 2019-04-28

**Authors:** Wafa Ali AlDhaleei, Akshaya Srikanth Bhagavathula, Rabia Aldoghaither

**Affiliations:** ^1^Internal Medicine Specialist, Sheikh Khalifa Medical City, P.O. Box: 51900, Abu Dhabi, UAE; ^2^Department of Internal Medicine, College of Medicine and Health Sciences, UAE University, P.O. Box: 15551, Al Ain, UAE; ^3^Infectious Diseases Consultant, Sheikh Khalifa Medical City, P.O. Box: 51900, Abu Dhabi, UAE

## Abstract

*Staphylococcus lugdunensis* is a coagulase-negative staphylococcus that leads to destructive infective endocarditis. The clinical course of *S. lugdunensis* endocarditis is usually aggressive with a high mortality rate compared to endocarditis caused by other coagulase-negative staphylococcal species. Despite that, it is usually sensitive to Penicillin G, and surgical intervention is sometimes warranted. Here, we report a case of *S. lugdunensis* endocarditis complicated by both embolic stroke and meningitis.

## 1. Introduction

*Staphylococcus lugdunensis* is a Gram-positive, coagulase-negative *Staphylococcus* and was first described by Freney et al. in 1988 [1]. *S. lugdunensis* endocarditis is usually destructive in a native as well as a prosthetic valve. The incidence of *S. lugdunensis* endocarditis varies from 3 to 7 per 100,000 person-years annually worldwide [2]. *S. lugdunensis* meningitis has been reported in the previous literature as a single pathology [3–5]. However, it is worth mentioning that we report the first case of *S. lugdunensis* native valve endocarditis which was complicated by both embolic stroke and meningitis.

## 2. The Case

A 44-year-old nonalcoholic and nonsmoker man, known polymyositis, dyslipidemia, and asthma, presented to the hospital with fever for ten days, associated with a left-side headache, dry cough, and also night sweats in the past two months. He also reported impaired sensation over the left side of the face and body with left-sided facial droop and left-sided weakness. The patient reported a weight loss of 7 Kg over the last two months. On the second day of fever, he was seen in a clinic and was given amoxicillin-clavulanic acid 1 g daily for seven days course, for possible otitis media. Despite this, his fever did not subside. Then, four days before the admission, he received cefixime 400 mg (3 doses only) after which he was admitted.

**Figure 1:**
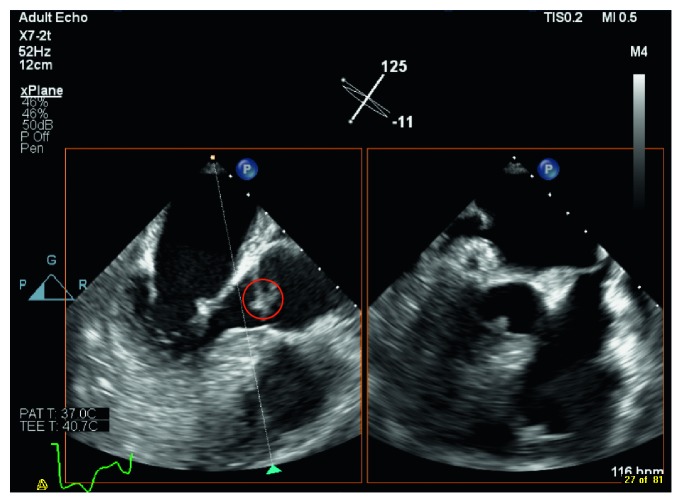
Aortic valve vegetation.

**Figure 2:**
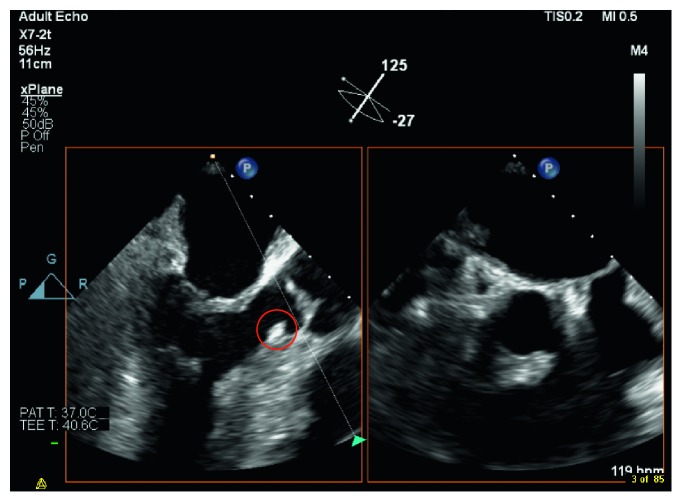
Subaortic valve vegetation.

**Figure 3:**
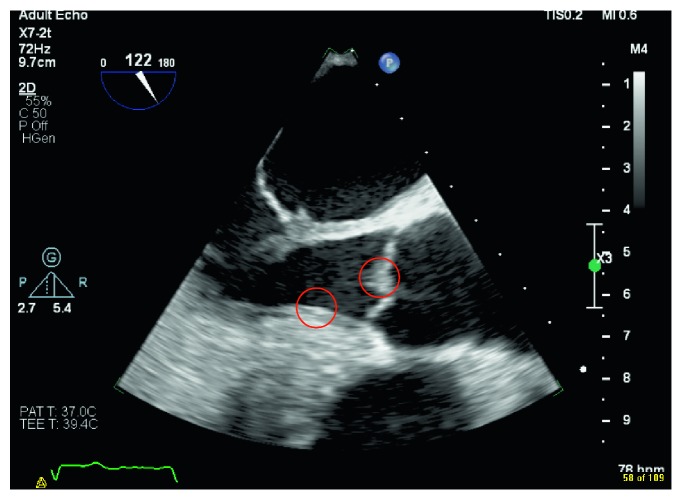
Aortic valve posttreatment.

Blood culture grew *S. lugdunensis* on the day of admission and day 3 of hospitalization, transthoracic echo (TTE) and transesophageal echo (TEE) identified vegetations on the aortic valve (reported to be < 1 cm), and myxomatous thickened mitral valve (possible vegetations) with moderate mitral regurgitation (Figures 1 and 2). He was started on vancomycin for the treatment of infective endocarditis. His recent neurological deficits were attributed to embolic stroke secondary to the infective endocarditis. Repeat blood culture on the fifth day of hospitalization was negative. On the third day of hospitalization, the patient complained of worsening headache, and the CT brain showed diffuse leptomeningeal, tentorium, and falx enhancement, with highly suspicious faint linear enhancement along the right central sulcus. Lumbar puncture was done the following day, and the cerebrospinal fluid (CSF) analysis revealed leukocytosis of neutrophils predominance of 47% with very high protein and very low glucose levels. Moreover, CSF culture grew *S. lugdunensis*. The patient received four days of vancomycin alone (loading dose of 1 g then adjusted according to the vancomycin trough level). Then, flucloxacillin 2 g q6 hrs was added to vancomycin for a total duration of 17 days. After that, the patient received flucloxacillin 2 g q6 hrs alone for an additional 27 days. Flucloxacillin was added to vancomycin, given the severity of the case presentation. The cardiothoracic team evaluated the patient, and they decided that surgical intervention is not indicated. Repeated TEE before discharge revealed aortic valve vegetation which is smaller in size (Figure 3). Upon discharge, he was able to ambulate with support and required outpatient rehabilitation follow-ups.

## 3. Discussion

We report the first case of *S. lugdunensis* endocarditis causing meningitis and embolic stroke. *S. lugdunensis* was first described in 1988 as a causative organism for endocarditis [6]. It can produce biofilms that enable it to adhere to prosthetic materials and native tissues [7]. *S. lugdunensis* endocarditis has also been reported in patients with cardiac implantable devices and prosthetic valves [8–11].

*S. lugdunensis* native valve endocarditis has been associated with various complications ranging from a myocardial abscess, septic emboli, and valve perforation [11]. Our patient had involvement of the aortic valve, but other valves such as mitral and tricuspid were also involved but to a lesser extent. Ishiekwene et al. also reported the involvement of the aortic valve with a ventricular septal defect [7]. However, Chung et al. reported the involvement of the tricuspid valve [12]. Kline et al. reported case series of different valve involvement [13].

The neurological manifestations secondary to septic embolization can range from altered sensation, weakness, and visual defects [14]. It appears that embolic stroke secondary to *S. lugdunensis* endocarditis is highly seen in middle-aged men and women [2]. However, *S. lugdunensis* meningitis has been reported in the previous literature as a complication postoperative and a single pathology without endocarditis [3–5]. Our patient presented with endocarditis complicated by both stroke and meningitis, in which meningitis has not been yet reported as a complication of *S. lugdunensis* endocarditis.

*S. lugdunensis* endocarditis has been associated with high mortality [15], although most of the *S. lugdunensis* isolates are sensitive to Penicillin G [13, 16]. High mortality is associated with the ability of the *S. lugdunensis* to cause tissue destruction leading to acute heart failure. Therefore, early surgical intervention has been associated with favorable outcomes in such cases [6, 12]. The efficacy of medical treatment versus a combination of medical and surgical treatment needs further investigation [2]. Our patient responded to medical treatment alone.

## 4. Conclusion

*S. lugdunensis* endocarditis is unusual. We reported a case of *S. lugdunensis* endocarditis complicated by both embolic stroke and meningitis. Careful evaluation and assessment are warranted to direct the appropriate therapy of patients.
